# NanoBiT Complementation to Monitor Agonist-Induced Adenosine A_1_ Receptor Internalization

**DOI:** 10.1177/2472555219880475

**Published:** 2019-10-04

**Authors:** Mark Soave, Barrie Kellam, Jeanette Woolard, Stephen J. Briddon, Stephen J. Hill

**Affiliations:** 1 Division of Physiology, Pharmacology and Neuroscience, School of Life Sciences, University of Nottingham Medical School, Queen’s Medical Centre, Nottingham, UK; 2 Centre of Membrane Proteins and Receptors (COMPARE), University of Birmingham and University of Nottingham, The Midlands, UK; 3 School of Pharmacy, Centre for Biomolecular Sciences, University of Nottingham, Nottingham, UK

**Keywords:** GPCR, adenosine, receptor internalization, NanoBiT, nanoluciferase complementation

## Abstract

Receptor internalization in response to prolonged agonist treatment is an important regulator of G protein–coupled receptor (GPCR) function. The adenosine A_1_ receptor (A_1_AR) is one of the adenosine receptor family of GPCRs, and evidence for its agonist-induced internalization is equivocal. The recently developed NanoBiT technology uses split NanoLuc Luciferase to monitor changes in protein interactions. We have modified the human A_1_AR on the N-terminus with the small high-affinity HiBiT tag. In the presence of the large NanoLuc subunit (LgBiT), complementation occurs, reconstituting a full-length functional NanoLuc Luciferase. Here, we have used complemented luminescence to monitor the internalization of the A_1_AR in living HEK293 cells. Agonist treatment resulted in a robust decrease in cell-surface luminescence, indicating an increase in A_1_AR internalization. These responses were inhibited by the A_1_AR-selective antagonist 1,3-dipropyl-8-cyclopentylxanthine (DPCPX), with an antagonist affinity that closely matched that measured using ligand binding with a fluorescent A_1_ receptor antagonist (CA200645). The agonist potencies for inducing A_1_AR internalization were very similar to the affinities previously determined by ligand binding, suggesting little or no amplification of the internalization response. By complementing the HiBiT tag to exogenous purified LgBiT, it was also possible to perform NanoBRET ligand-binding experiments using HiBiT–A_1_AR. This study demonstrates the use of NanoBiT technology to monitor internalization of the A_1_AR and offers the potential to combine these experiments with NanoBRET ligand-binding assays.

## Introduction

G protein–coupled receptors (GPCRs) are the largest family of membrane-signaling proteins and are able to modulate signals from a wide range of endogenous ligands.^1^ Prolonged stimulation by an agonist results in the internalization of many GPCRs, and this process can occur via different pathways, including caveolae-dependent and clathrin-mediated processes.^2,3^ For the latter, G protein–coupled receptor kinases (GRKs) phosphorylate serine and threonine residues within the intracellular loops and C-terminal tail of the receptor following agonist-stimulated receptor activation.^4^ β-Arrestins are able to bind to the phosphorylated receptor and can initiate downstream signaling pathways that are independent of G proteins.^5^ β-Arrestins also compete sterically with G proteins for binding to the receptor, resulting in receptor desensitization, and recruit specific adaptor proteins that are required for clathrin-mediated endocytosis.^6^ The GPCRs are internalized in clathrin-coated vesicles and transferred into early endosomes, where it is now known that a second wave of intracellular signaling can occur.^7,8^

The adenosine A_1_ receptor (A_1_AR) is part of the wider adenosine GPCR subfamily, grouped by their ability to bind their endogenous ligand, adenosine.^1,9^ The A_1_AR predominantly couples to the G_i_ family of heterotrimeric G proteins, which inhibit adenylyl cyclase–mediated cAMP (cyclic adenosine monophosphate) production. There is contrasting evidence, however, concerning the nature of GRK-mediated A_1_AR phosphorylation,^10–13^ as well as the nature and extent of A_1_AR internalization in response to chronic stimulation by agonists.^12–15^ The A_1_AR is able to internalize through both clathrin- and caveolae-dependent endocytosis.^13,16^ Previous studies of human A_1_AR internalization observed that the receptor had a slow rate of internalization of several hours.^15,17–19^ Ruiz et al. found this was also true of the rat A_1_AR receptor, which required more than 12 h of stimulation to internalize 50% of rat A_1_ARs in cortical neurons.^20^ These A_1_AR data contrast drastically with data for the other G_i_-coupled adenosine receptor, the A_3_ receptor, which internalizes more rapidly, and within minutes of agonist stimulation.^12,21,22^

Previous studies on A_1_AR internalization have been conducted using either radiolabeled A_1_AR ligands^14,15^ or confocal microscopy.^11,19^ These techniques offer specific advantages, such as the ability to monitor A_1_AR internalization in ex vivo tissues with radioligand binding, or the ability to directly visualize internalization with microscopy. These methods are, however, intensive and low throughput.

Recently, NanoLuc Binary Technology (NanoBiT; Promega, Southampton, UK) has been developed that splits the bright NanoLuc Luciferase^23^ into two segments, a large 18 kDa fragment (termed LgBiT) and a much smaller 1.3 kDa fragment (termed SmBiT; a small complementation tag).^24^ These fragments have low intrinsic affinity for each other [equilibrium dissociation constant (K_D_), 190 µM] and complement to form the full bioluminescent protein NanoLuc. SmBiT–LgBiT complementation has successfully been used to monitor protein–protein interactions of membrane receptors, including the recruitment of G proteins and β-arrestins to GPCRs.^25,26^ In the development of the NanoBiT system, other small complementary peptides were identified that have different affinities for LgBiT. One short, 11-amino-acid sequence had a very high affinity for LgBiT (K_D_ = 700 pM; termed HiBiT^24^). As an 18 kDa fragment, the LgBiT is cell impermeable, and therefore HiBiT–LgBiT complementation provided an approach to distinguish between internalized proteins and those retained at the cell surface. This would not have been possible with the full-length NanoLuc Luciferase. In this study, we have used A_1_ARs tagged on the N-terminus with HiBiT to determine whether NanoBiT complementation can be used as a high-throughput method to monitor loss of A_1_ARs from the cell surface in living cells.

## Materials and Methods

### Materials

Adenosine and 5′-*N*-ethylcarboxamidoadenosine (NECA) were purchased from Sigma-Aldrich (Gillingham, UK). 1,3-Dipropyl-8-cyclopentylxanthine (DPCPX), 2-chloro-*N*^6^-cyclopentyladenosine (CCPA), 2′methyl-2-chloro-*N*^6^-cyclopentyladenosine (2-MeCCPA), and 2-phenylaminoadenosine (CV-1808) were obtained from Tocris (Bristol, UK). 2-Amino-6-[[2-(4-chlorophenyl)-1,3-thiazol-4-yl]methylsulfanyl]-4-[4-(2-hydroxyethoxy)phenyl]pyridine-3,5-dicarbonitrile (capadenoson) was purchased from Haoyuan Chemexpress (Shanghai, China). The fluorescent antagonist CA200645 was purchased from HelloBio (Bristol, UK). Purified LgBiT, restriction enzymes, FuGENE HD Transfection Reagent, and furimazine were purchased from Promega.

### Constructs and Cell Lines

To create the HiBiT–A_1_AR construct, the full-length NanoLuc sequence was removed from the pcDNA3.1 NLuc–A_1_AR vector^27^ using KpnI and BamHI restriction sites. This left the pcDNA3.1 vector containing the A_1_AR with a mutated start codon (Met → Leu). Primers containing the HiBiT sequence (bold letters), a GSSGGSSG linker (5′: c**ATGGTGAGCGGCTGGCGGCTGTTCAAGAAGATTAGC**GGGAGTTCTGGCGGCTCGAGCGGTg; and 5′: gatccACCGCTCGAGCCGCCAGAACTCCC**GCTAATCTTCTTGAACAGCCGCCAGCCGCTCACCATggtac**), and the respective KpnI and BamHI overhangs (lowercase letters) were phosphorylated using T4 Polynucleotide Kinase (NEB, Hitchin, UK) and annealed for 30 min at 37 °C. The annealed primers were then ligated into the digested pcDNA3.1 A_1_AR vector using T4 ligase (NEB), creating the full-length fusion protein HiBiT–A_1_AR. Correct insertion was confirmed by DNA sequencing using the School of Life Sciences Sequencing Facility at the University of Nottingham.

HEK293 cells were maintained in Dulbecco’s modified Eagle medium (DMEM) containing 10% fetal calf serum (FCS) and 2 mM L-glutamine at 37 °C in a 5% CO_2_ atmosphere. A mixed-population HiBiT–A_1_AR stable cell line was generated using FuGENE HD (Promega), according to the manufacturer’s instructions, and the cells were subjugated to 3 weeks of selection with 1 mg/mL G418.

### NanoBiT Internalization Assay

HEK293 cells stably expressing HiBiT–A_1_AR were plated onto white 96-well plates (Greiner Bio-One, Monroe, NC) previously coated with 10 µg/mL poly-D-lysine. 100 µL DMEM containing cells in suspension (30,000 cells/well) was added to each well, and the plate incubated at 37 °C in a 5% CO_2_ atmosphere for 24 h. The next day, the medium was removed from each well and replaced with 50 µL HEPES-buffered saline solution (HBSS; 145 mM NaCl, 5 mM KCl, 10 mM HEPES, 1.3 mM CaCl_2_ dihydrate, 1.5 mM NaHCO_3_, 2 mM sodium pyruvate, 1 mM MgSO_4_.7H_2_O, 10 mM D-glucose; pH 7.45) and the relevant concentration of ligand. For end-point assays, cells were incubated at 37 °C for 2 h. Purified LgBiT was diluted in HBSS (10 nM final concentration) and added to each well in the presence of furimazine (1:400 final concentration). The plate was incubated for 15 min in the dark at 37 °C, allowing complementation to occur. Luminescence was measured on the PHERAstar FS plate reader (BMG Labtech, Offenburg, Germany) using the LUM Plus module.

### Bioluminescence Imaging

HEK293 cells stably expressing HiBiT–A_1_AR were seeded onto a poly-D-lysine-coated (10 µg/mL) 35 mm four-chamber MatTek dish (Ashland, MA) at a density of 120,000 cells/mL. The dish was incubated at 37 °C in a 5% CO_2_ atmosphere for 24 h. The next day, the medium was removed and replaced with 400 µL HBSS containing furimazine (1:400 final concentration). Purified LgBiT was added, and the plate incubated at 37 °C for 20 min. Bright-field and bioluminescence imaging was performed on the Olympus LV200 inverted microscope (Olympus, Southend, UK). Bright-field images were captured with a 50 ms exposure. Bioluminescence images were captured with a 45 s exposure, using a Hamamatsu EM-CCD (electron-multiplying charge-coupled device; Hamamatsu, Hamamatsu City, Japan) with a gain of 100.

### NanoBRET Ligand-Binding Assay

HEK293 HiBiT–A_1_AR cells were seeded onto poly-D-lysine-coated white 96-well plates as described above. The next day, the medium was removed from each well and replaced with 50 µL HBSS containing 10 nM LgBiT; the plate was incubated for 15 min in the dark at 37 °C, allowing complementation to occur. The HBSS with unbound LgBiT was removed and replaced with 50 µL HBSS containing the fluorescent A_1_ receptor antagonist ligand CA200645^28^ in the absence or presence of 10 µM DPCPX. The plate was incubated in the dark at 37 °C for 2 h. Furimazine (1:400 final concentration) was added to each well, and the plate incubated for 15 min at 37 °C. The resulting bioluminescence resonance energy transfer (BRET) was measured using the PHERAstar FS plate reader (BMG Labtech), which simultaneously measured filtered light emissions at 460 nm (80 nm bandpass) and >610 nm (longpass). The BRET ratio was calculated by dividing the >610 nm emission by the 460 nm emission.

### Data Analysis

Data were presented and analyzed using Prism 7 software (GraphPad, San Diego, CA).

The potency of ligands that internalized HiBiT–A_1_AR was determined from fitting data to a one-site sigmoidal concentration–response curve defined by the following three-parameter logistic equation:

(1) $$\%\;\;receptor\;at\;cell\;surface=100-\frac{\left(100\times \left[A^{n}\right]\right)}{\left(\left[A^{n}\right]+IC_{50}^{n}\right)}$$

where [*A*] is the concentration of the ligand *n* is the Hill coefficient, and *IC*_50_ is the concentration of ligand required to internalize 50% of receptors.

In the experiments in which three fixed concentrations of DPCPX were used, the K_D_ of DPCPX was estimated from the shift in the NECA response by 10 nM DPCPX using the Gaddum equation:^29^

(2) $$\mathrm{CR}=1+\frac{\left[B\right]}{K_{B}}$$

where *CR* is the concentration ratio of NECA required to stimulate an identical response in the presence or absence of 10 nM DPCPX [*B*], and *K_B_* is the affinity of DPCPX.

The time course of internalization in response to 10 µM NECA at HiBiT–A_1_AR was fitted with a one-phase exponential decay curve using the following equation:

(3) $$Y={\left(Y_{0}-NS\right)}^{-k.t}+NS$$

where *Y* is the luminescence at time *t* minutes, *Y*_0_ was the luminescence at time 0, *NS* is the background luminescence, and *k* is the rate constant of the decrease in luminescence per minute.

Saturation NanoBRET experiments were simultaneously fitted to obtain the total and nonspecific components using the following equation:

(4) $$BRET\;Ratio=\frac{B_{max}\times \left[B\right]}{\left[B\right]+K_{D}}+\left(\left(M\times \left[B\right]\right)+C\right)$$

where *B_max_* is the maximal level of specific binding, [*B*] is the concentration of fluorescent ligand in nM, *K_D_* is the equilibrium dissociation constant, *M* is the slope of the linear nonspecific binding component, and *C* is the *y*-axis intercept.

Data are presented as the mean ± SEM of triplicate determinations in a single experiment. In the text, *n* refers to the number of separate experiments. Statistical significance was defined as *p* < 0.05 using Student’s unpaired *t* test.

## Results

**Figure 1.**
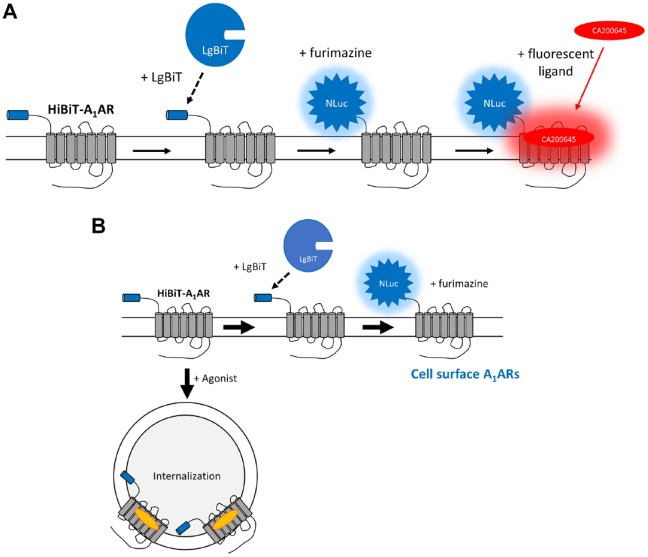
(**A**) Schematic of the use of NanoLuc Binary Technology (NanoBiT) complementation to monitor the expression of a high-affinity tag–adenosine A_1_ receptor (HiBiT–A_1_AR) at the cell surface. A_1_ARs tagged with HiBiT on their N-terminus are expressed at the cell surface of HEK293 cells. On addition of exogenous purified LgBiT (the large NanoLuc subunit), complementation occurs between HiBiT and LgBiT, forming the full-length NanoLuc (NLuc) Luciferase. In the presence of the substrate furimazine, the complemented NLuc is luminescent. Addition of a red A_1_ receptor fluorescent ligand, such as CA200645, that binds to the HiBiT-tagged A_1_AR allows NanoBRET to occur between the NLuc and the fluorescent ligand. (**B**) Schematic of the NanoBiT internalization assay. Under basal conditions, N-terminally HiBiT-tagged A_1_ARs are expressed on the plasma membrane of HEK293 cells. On addition of exogenous purified LgBiT, complementation occurs between HiBiT and LgBiT, forming the full-length NLuc Luciferase. In the presence of the substrate furimazine, luminescence is read as the experimental output. When treated with an agonist, the HiBiT–A_1_AR undergoes internalization and is removed from the plasma membrane. After 2 h, purified LgBiT is added to the medium, but it cannot cross the plasma membrane and complement with the HiBiT–A_1_AR, and therefore there is less complemented NLuc as a result.

NanoBiT has provided a platform for assessing protein–protein interactions in vitro in real time.^24,30–32^ Here, we have used the NanoBiT complementation technology to monitor the presence of the human A_1_ receptor, tagged on its N-terminus with HiBiT, at the cell surface of living cells following addition of purified LgBiT (**Fig. 1a**). This approach can also be extended to detect the loss of HiBiT–A_1_AR following agonist stimulation as a method to detect receptor internalization (**Fig. 1b**).

**Figure 2.**
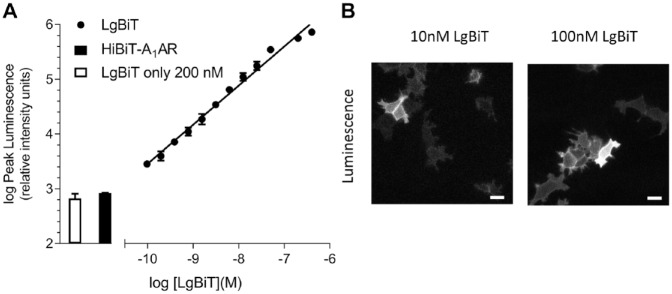
(**A**) The effect of purified LgBiT (the large NanoLuc subunit) titrations on complemented luminescence in HEK293 cells stably expressing the high-affinity tag–adenosine A_1_ receptor (HiBiT–A_1_AR) following a 15 min incubation with furimazine (1:400). Bars indicate the luminescence measured by 200 nM LgBiT (open bar) and HiBiT–A_1_AR (closed bar) alone, respectively. Data are mean ± SEM from triplicate determinations in a single experiment. This single experiment is representative of four independent experiments performed. (**B**) Wide-field bioluminescence imaging of HEK293 cells stably expressing HiBiT–A_1_AR with increasing concentrations of purified LgBiT. Cells were incubated with LgBiT and furimazine (1:400) for 20 min prior to imaging. Images are representative of three separate experiments. Scale bars show 20 µm.

Expression of the HiBiT–A_1_AR at the plasma membrane was first confirmed using NanoBiT complementation. HEK293 cells, stably transfected with HiBiT–A_1_AR, were incubated with purified LgBiT in the presence of the NanoLuc substrate, furimazine. As an 18 kDa protein, LgBiT is cell impermeable and thus will complement only with HiBiT–A_1_AR present on the plasma membrane. Increasing concentrations of LgBiT resulted in a higher luminescence signal (**Fig. 2a**; *n* = 4). These results indicated that HiBiT–A_1_AR was able to traffic to the plasma membrane and complement with exogenously applied purified LgBiT. It should be noted that neither HiBiT–A_1_AR nor LgBiT alone produced a strong luminescent signal (**Fig. 2a**). Wide-field bioluminescent imaging also confirmed clear membrane expression of HiBiT–A_1_AR in HEK293 cells (**Fig. 2b**). From these experiments, it was determined that 10 nM LgBiT would provide a sufficient luminescence response window for all subsequent assays.

**Figure 3.**
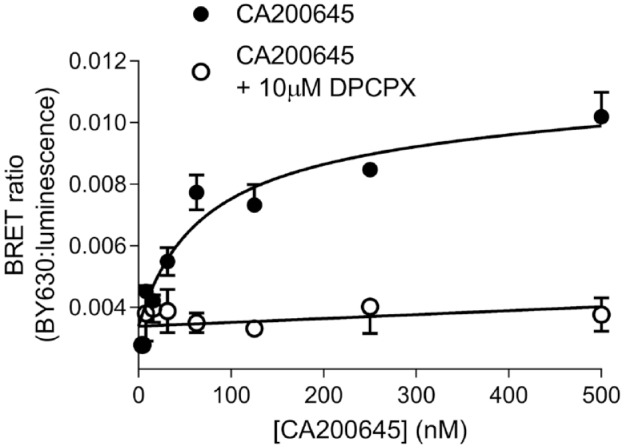
NanoBRET ligand binding at the adenosine A_1_ receptor (A_1_AR) using fully complemented HiBiT–LgBiT (high-affinity tag–large NanoLuc subunit). 10 nM LgBiT was first added to HEK293 cells stably expressing HiBiT–A_1_AR before addition of increasing concentrations of CA200645 for 2 h. Nonspecific binding was determined in the presence of 10 µM 1,3-dipropyl-8-cyclopentylxanthine (DPCPX). Data are mean ± SEM from triplicate determinations in a single experiment. This single experiment is representative of four independent experiments performed.

To confirm that the complementation of HiBiT–A_1_AR with exogenously applied LgBiT did not alter the ability of the A_1_AR to bind ligands, ligand-binding studies were performed using NanoBRET with a fluorescent A_1_ antagonist^28,33^ (**Fig. 1a**). Following complementation of HiBiT–A_1_AR with purified LgBiT, the binding of the fluorescent A_1_ receptor antagonist CA200645 to the complemented A_1_ receptor was monitored using NanoBRET^28^ (**Figs. 1 and 3**). In these assays, clear specific binding of CA200645 to the A_1_AR was observed. The negative log of the dissociation constant (pK_D_) of CA200645 was calculated to be 7.17 ± 0.03 (K_D_ = 63.8 nM; *n* = 4; **Fig. 3**). This was similar to the K_D_ value reported previously (K_D_ = 33.8 nM) for CA200645 to the full-length NanoLuc-tagged A_1_AR.^27^

**Figure 4.**
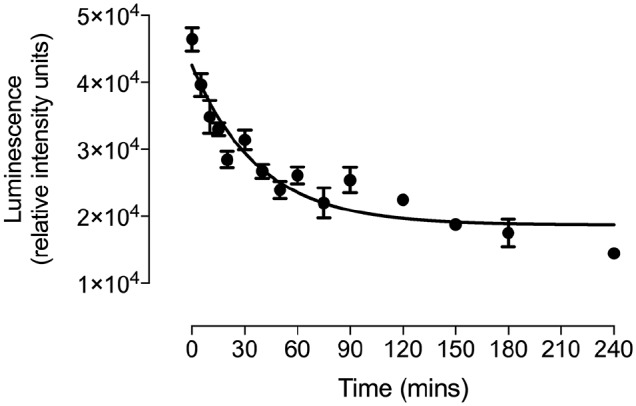
Time course of high-affinity tag–adenosine A_1_ receptor (HiBiT–A_1_AR) internalization in HEK293 cells. Luminescence of cell surface complemented HiBiT–LgBiT (large NanoLuc subunit) in cells treated with 10 µM 5′-*N*-ethylcarboxamidoadenosine (NECA) for up to 4 h. Data are mean ± SEM of triplicate determinations from a single experiment. This single experiment is representative of five separate experiments.

With successful membrane expression of HiBiT–A_1_AR confirmed, it was then established if NanoBiT could be used to monitor receptor internalization (**Fig. 1b**). Cells were incubated at 37 °C and treated with 10 µM NECA for increasing periods of time to stimulate an internalization response. Longer incubation periods resulted in a decrease in luminescence (**Fig. 4**), correlating to an increase in the proportion of HiBiT–A_1_ARs that have internalized (**Fig. 1b**). The resulting half-life of internalization was 31 ± 6 min (*n* = 4). From these data, a 2 h ligand incubation time was chosen for all subsequent end-point experiments, because receptor internalization had plateaued by this point.

**Figure 5.**
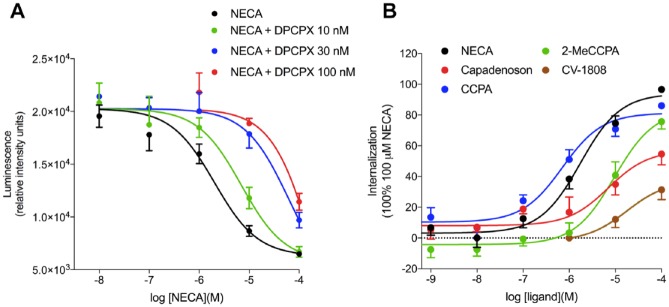
(**A**) Ability of 1,3,-dipropyl-8-cyclopentylxanthine (DPCPX) to inhibit the NECA-induced internalization response in the high-affinity tag–adenosine A_1_ receptor (HiBiT–A_1_AR). Internalization of HiBiT–A_1_AR in response to 5′-*N*-ethylcarboxamidoadenosine (NECA) in the absence or presence of the antagonist DPCPX at 10, 30, and 100 nM, respectively. Data are mean ± SEM from triplicate determinations in a single experiment. This single experiment is representative of six separate experiments. (**B**) Concentration–response curves of HiBiT–A_1_AR internalization stimulated by a panel of A_1_ receptor agonists in HEK293 cells. Internalization is reported as a percentage of internalization stimulated by a 2 h incubation with 100 µM NECA. Data are mean ± SEM from at least five separate experiments, in which each experiment was performed in triplicate. The number of individual experiments performed with each agonist is given in **Table 1**.

NECA stimulated a concentration-dependent loss of HiBiT–A_1_ARs from the cell surface of HEK293 cells (pIC_50_ = 5.67 ± 0.21; *n* = 10; **Fig. 5a and Table 1**). The A_1_-selective antagonist DPCPX was able to inhibit the NECA-stimulated internalization response (**Fig. 5a**). Fitting the Gaddum equation to the responses measured in the presence and absence of 10 nM DPCPX produced an affinity of DPCPX (pK_D_ = 8.28 ± 0.12; *n* = 6), which closely matches the affinity of DPCPX calculated in the human NLuc–A_1_AR.^27^

**Table 1.** pEC_50_ and E_max_ Values of Agonist-Induced Internalization of HiBiT–A_1_AR in HEK293 Cells.

Ligand	Internalization pEC_50_ (Mean ± SEM)	E_max_ (% 100 µM NECA)	*n*
NECA	5.67 ± 0.21	100	10
Capadenoson	5.23 ± 0.32	57.8 ± 4.4^†^	5
CCPA	6.43 ± 0.18	84.4 ± 6.5	10
2-MeCCPA	4.96 ± 0.13	86.7 ± 4.1	5
CV-1808	4.65 ± 0.24	47.1 ± 17.1^†^	5

Data are mean ± SEM from *n* separate experiments. †Internalization stimulated by the highest concentration (100 µM) of this ligand (*p* < 0.05, comparing Emax vs. that of 100 µM NECA). A1AR, adenosine A1 receptor; CCPA, 2-chloro-*N*6-cyclopentyladenosine; HiBiT, a high-affinity NanoLuc Luciferase tag; NECA, 5′-*N*-ethylcarboxamidoadenosine.

A panel of adenosine A_1_ receptor agonists were screened for their ability to internalize HiBiT–A_1_AR. In addition to NECA, the A_1_AR agonists CCPA and 2-MeCCPA were found to be full agonists in this assay, able to stimulate a robust internalization of HiBiT–A_1_AR (**Fig. 5b and Table 1**). The atypical A_1_AR agonist capadenoson and the adenosine A_2_ receptor ligand CV-1808 were found to elicit partial internalization responses (**Fig. 5b and Table 1**).

## Discussion

NanoBiT has provided the opportunity to measure GPCR–effector interactions in real time in living cells with a high degree of sensitivity.^25,26,31,32^ Here, we have used the high-affinity HiBiT tag to detect cell surface expression of the adenosine A_1_ receptor and quantified the loss of receptors at the cell surface in response to agonist treatment as a measure of receptor internalization.

The HiBiT–A_1_AR was successfully expressed on the surface of HEK293 cells and could be visualized with bioluminescent imaging following addition of exogenous purified LgBiT. Furthermore, the complemented NLuc–A_1_AR retained the ability to bind fluorescent adenosine A_1_AR antagonists, yielding binding constants that were similar to those determined previously using A_1_ARs expressing the full-length NLuc tag.^27,28^ The strong luminescence signal provided by fully complemented HiBiT–LgBiT, however, provided a large assay window to detect small changes in the surface expression of the A_1_AR. This can be observed in **Figure 4** as the loss of luminescence in response to increasing incubation periods with the agonist NECA.

The human adenosine A_1_ receptor has been shown to internalize in recombinant cell lines using radioligand binding.^15,17,18^ These studies observed a slow rate of A_1_AR internalization, with a *t*_1/2_ for internalization of several hours. Using NanoBiT, it was possible to detect A_1_AR internalization (**Fig. 4**) at much earlier time points than was previously reported.^17–19^ In addition, both radioligand binding and confocal microscopy are low-throughput techniques and are not amenable to performing full concentration–response curves for a panel of A_1_AR agonists.

In contrast, NanoBiT provided an ideal platform for detecting the internalization of A_1_AR in living cells in response to a wide panel of ligands. The high signal-to-noise ratio of the assay made it possible to monitor full-agonist and partial-agonist responses during relatively short periods of time (**Fig. 5b**). The potencies of the agonist responses were generally similar to the affinities of the ligands at the human A_1_AR, as measured previously with NanoBRET.^27^ Thus, the similarity in values obtained for NECA and CCPA for the two assays suggests that there is no signal amplification in the internalization response for these two agonists. This contrasts with the higher receptor–effector coupling observed for these ligands for cAMP inhibition or pERK1/2 phosphorylation.^34,35^

The one exception was the partial A_1_AR agonist capadenoson,^36,37^ in which the pEC_50_ (5.23) for internalization was more than an order of magnitude lower than its pKi value (6.85), determined from inhibition of NanoBRET binding with CA200645 at the human A_1_AR.^27^ This very low potency for internalization of capadenoson compared to its binding affinity for the orthosteric ligand-binding site suggests that there may be a more complex mechanism of action involved. This may involve differential affinities for multiple A_1_AR agonist receptor conformations and the potential for signaling bias.^35,38,39^

The internalization stimulated by NECA could be antagonized by the A_1_AR antagonist DPCPX in a concentration-dependent manner. The resulting analysis suggested an affinity for DPCPX that was in keeping with the known affinity of this ligand for the human A_1_AR.^27,40^ In addition, there was no hint of inverse agonism with DPCPX in this assay (**Table 1**).

These experiments were performed in HEK293 cells, which express both adenosine A_2A_ and A_2B_ receptors endogenously.^41^ It is unlikely that the internalization responses measured in this study were affected by the presence of the adenosine A_2A_ or A_2B_ receptors, given that the potencies of the ligands used in this study are in the same rank order as the binding affinities of the human A_1_AR,^27,40,42^ and the determined affinity for DPCPX was in keeping with the known affinity at the human A_1_AR.

It should be noted that the assay described here has been configured specifically to monitor the extent of loss of A_1_ receptors from the cell surface in response to agonist treatment. From the data obtained, we cannot comment on the extent to which A_1_ agonists also alter A_1_ receptor protein degradation and turnover. It should be noted, however, that NanoBiT represents a versatile technology with a broad dynamic range that can be applied to detect and quantify protein expression^30^ and degradation.^43^ For example, Riching et al. have exploited the high sensitivity of HiBiT–LgBiT complementation to detect targets with low levels of native expression and measure their subsequent degradation following PROTAC treatment.^43^ A similar approach using a HiBiT tag on the C-terminus of the A_1_ receptor and its expression in cells that also express cytosolic LgBiT would allow A_1_ receptor turnover and degradation to be monitored.

In conclusion, this study reports the use of NanoBiT to monitor A_1_AR internalization in a plate-based assay based on complemented nanoluciferase luminescence. This approach can readily be applied to other GPCRs or indeed any cell surface membrane receptors (e.g., receptor tyrosine kinases or cytokine receptors) through the introduction of the HiBiT tag at the extracellular terminus of the protein of interest.
